# Mesenteric panniculitis: systematic review of cross-sectional imaging findings and risk of subsequent malignancy

**DOI:** 10.1007/s00330-016-4298-2

**Published:** 2016-04-05

**Authors:** Steve Halligan, Andrew Plumb, Stuart Taylor

**Affiliations:** Centre for Medical Imaging, University College London UCL, 3rd Floor East, 250 Euston Road, London, NW1 2PG UK

**Keywords:** Panniculitis, Panniculitis, peritoneal, Sclerosing mesenteritis, Helical computed tomography, Computed tomography, spiral

## Abstract

**Objectives:**

Systematic review to determine any association between imaging features of idiopathic mesenteric panniculitis (MP) and subsequent malignancy.

**Methods:**

Two researchers searched primary literature independently for imaging studies of MP. They extracted data focusing on methodology for unbiased patient accrual and capability to determine a link between MP and subsequent malignancy. They noted imaging features of MP. Data were accrued and meta-analysis intended.

**Results:**

Fourteen of 675 articles were eligible; 1,226 patients. Only three (21 %) accrued patients prospectively. Twelve (86 %) studies described CT features. Follow-up varied widely; 1 month to 8 years. Prevalence of MP was influenced by accrual: 0.2 % for keyword search versus 1.7 % for consecutive series. Accrual bias affected nine (64 %) studies. 458 (38 %) of 1,209 patients had malignancy at accrual but varied widely (8–89 %), preventing meta-analysis. Sixty (6.4 %) of 933 patients developed new malignancy subsequently, also varying widely (0–11 %). Of just four studies that determined the proportion of unselected, consecutive patients with MP developing subsequent malignancy, three were retrospective and the fourth excluded patients with lymphadenopathy, likely excluding patients with MP.

**Conclusion:**

Studies were heterogeneous, with biased accrual. No available study can determine an association between MP and subsequent malignancy with certainty.

**Key Points:**

• *Our systematic review of mesenteric panniculitis found that imaging studies were biased*.

• *Spectrum and recruitment bias was largely due to retrospective study designs*.

• *No study could confirm a certain link between mesenteric panniculitis and subsequent malignancy*.

• *Excessive methodological heterogeneity precluded meaningful meta-analysis*.

• *High-quality research linking mesenteric panniculitis imaging features and subsequent malignancy is needed*.

## Introduction

Mesenteric panniculitis (MP) [1] describes inflammation of mesenteric fat and is manifest on CT scanning as a circumscribed region of increased mesenteric density, often exhibiting a mass-like effect, containing several lymph nodes, and engulfing mesenteric vessels [2, 3]. MP is known by a variety of alternative terms including ‘mesenteric lipodystrophy’ [4], ‘liposclerotic mesenteritis’ [5] and ‘misty mesentery’ [6, 7]. The latter term includes entities such as ‘sclerosing mesenteritis’ [8] and ‘retractile mesenteritis’ [9]. Such varied terminology reflects confusion regarding the precise definition of MP.

An association between MP and development of subsequent malignancy has been raised [10, 11], suggesting MP is an important finding. Alternatively, others state there is no association between MP and malignancy [12]. Lack of consensus regarding the clinical significance of MP thus presents radiologists with a diagnostic dilemma because MP is encountered frequently as an apparently incidental finding on cross-sectional imaging, usually abdominopelvic CT. Furthermore, referring clinicians will usually be unfamiliar with MP and will therefore look to the reporting radiologist for management guidance. To attempt to clarify the significance of MP in the face of apparently contradictory evidence we performed a systematic review of available medical literature, focusing on the cross-sectional imaging findings of MP and any association with development of subsequent malignancy.

## Methods

Ethical approval is not required by our institution for secondary research using primary literature.

### Data sources and search strategy

We wished to identify primary studies describing imaging findings of MP. To be as inclusive as possible we used all of the following terms: mesenteric panniculitis, mesenteritis, sclerosing panniculitis, sclerosing mesenteritis, misty mesentery, retractile mesenteritis, liposclerotic mesenteritis and mesenteric lipodystrophy. We combined terms in the following search string, to identify studies describing peritoneal manifestations:

- ((("mesentery"[MeSH Terms] OR "mesentery"[All Fields] OR "mesenteric"[All Fields]) OR sclerosing[All Fields] OR misty[All Fields] OR liposclerotic[All Fields] OR retractile[All Fields] OR ("peritoneum"[MeSH Terms] OR "peritoneum"[All Fields] OR "peritoneal"[All Fields])) AND (mesenteritis[All Fields] OR ("panniculitis"[MeSH Terms] OR "panniculitis"[All Fields]) OR ("lipodystrophy"[MeSH Terms] OR "lipodystrophy"[All Fields]))) OR (misty[All Fields] AND ("mesentery"[MeSH Terms] OR "mesentery"[All Fields]))

This string was then used to search the US National Library of Medicine PUBMED journal citation database (http://www.ncbi.nlm.nih.gov/pubmed). Results were then limited to humans (using: AND "humans"[MeSH Terms]), limited to English language (using: AND English[lang]), and case reports were excluded (using: NOT Case Reports[ptyp]). We used the systematic review filter (AND pmh_sr[sb]) to identify prior systematic reviews and reviewed the initial search (i.e. excluding filters) to identify any articles excluded incorrectly.

Two radiologists performed the search, one (SH) with 13 years’ experience of systematic review design, extraction and analysis, the other (AP) with 3 years’ experience. Disagreement was resolved face-to-face.

### Inclusion/exclusion criteria for primary studies

The electronic abstract of identified studies was read and the following exclusion criteria applied: We excluded articles without a focus on idiopathic MP. For example, we excluded IgG disease, post-renal transplant panniculitis, Nasu-Hakola disease and cutaneous panniculitis. Because we wished to investigate any association between MP and subsequent malignancy, we excluded studies exclusively recruiting patients known to have abdominopelvic malignancy or to have received abdominopelvic irradiation. Studies combining patients with and without malignancy were included. We excluded studies not reporting cross-sectional imaging data (we included ultrasound). Specifically, we excluded articles published before the introduction of CT scanning (i.e. pre 1973). We excluded narrative reviews and letters/correspondence. We excluded case reports and small cases series since these would not contribute sufficient unbiased data able to answer our research question. After consensus, we defined ‘small’ as ten or fewer cases recruited from a single centre. We defined single centre as a single hospital or single hospital grouping because we would expect such groupings to exhibit correlated patients and/or practice.

If no electronic abstract were available, or if a confident decision regarding exclusion was not possible, the full article was retrieved online and read. An excluded study log was kept, recording the single most relevant reason for exclusion (although we anticipated some articles would be rejected for multiple reasons), and passed to the other authors for review.

### Data extraction

The following data were extracted from full articles meeting inclusion criteria by the two radiologists performing the search (working independently), into a database (Excel for Mac 2011, Microsoft, Redmond WA): We noted sample size, gender and age distribution of participants, whether the study was single or multicentre, and whether accrual was prospective or retrospective. We noted the proportion of patients with malignancy and/or a history of abdominopelvic surgery at accrual, the proportion who were symptomatic, the length of follow-up and the proportion developing new malignancy during follow-up, and its nature. We assessed whether patient selection was subject to spectrum bias (e.g. by using a retrospective keyword search) and whether the study design allowed estimation of the proportion of patients without malignancy who then developed malignancy subsequent to their diagnosis of MP. We noted the imaging test used for diagnosis of MP and whether an independent reference test was adopted.

Regarding CT features of MP, we noted the following eight features: Proportion of patients exhibiting increased mesenteric density; whether a mesenteric ‘mass’ was present; linear dimensions of any mass; whether a ‘capsule’ was present [13]; the proportion of patients with lymphadenopathy; the largest nodal diameter; diameter range; proportion of patients with a nodal ‘halo’ [13].

### Analysis

Tabulated data were described by median and range (or mean where this was presented in the primary article). We wished to perform a meta-analysis to obtain a point-estimate describing the proportion of patients with MP who developed subsequent malignancy. Ultimately, meta-analysis was prevented by the small number of studies retrieved that presented adequate data, combined with excessive methodological heterogeneity, meaning that any point estimate would lack precision. We wrote our report noting PRISMA guidelines for transparent reporting of systematic reviews [14].

## Results

**Fig. 1:**
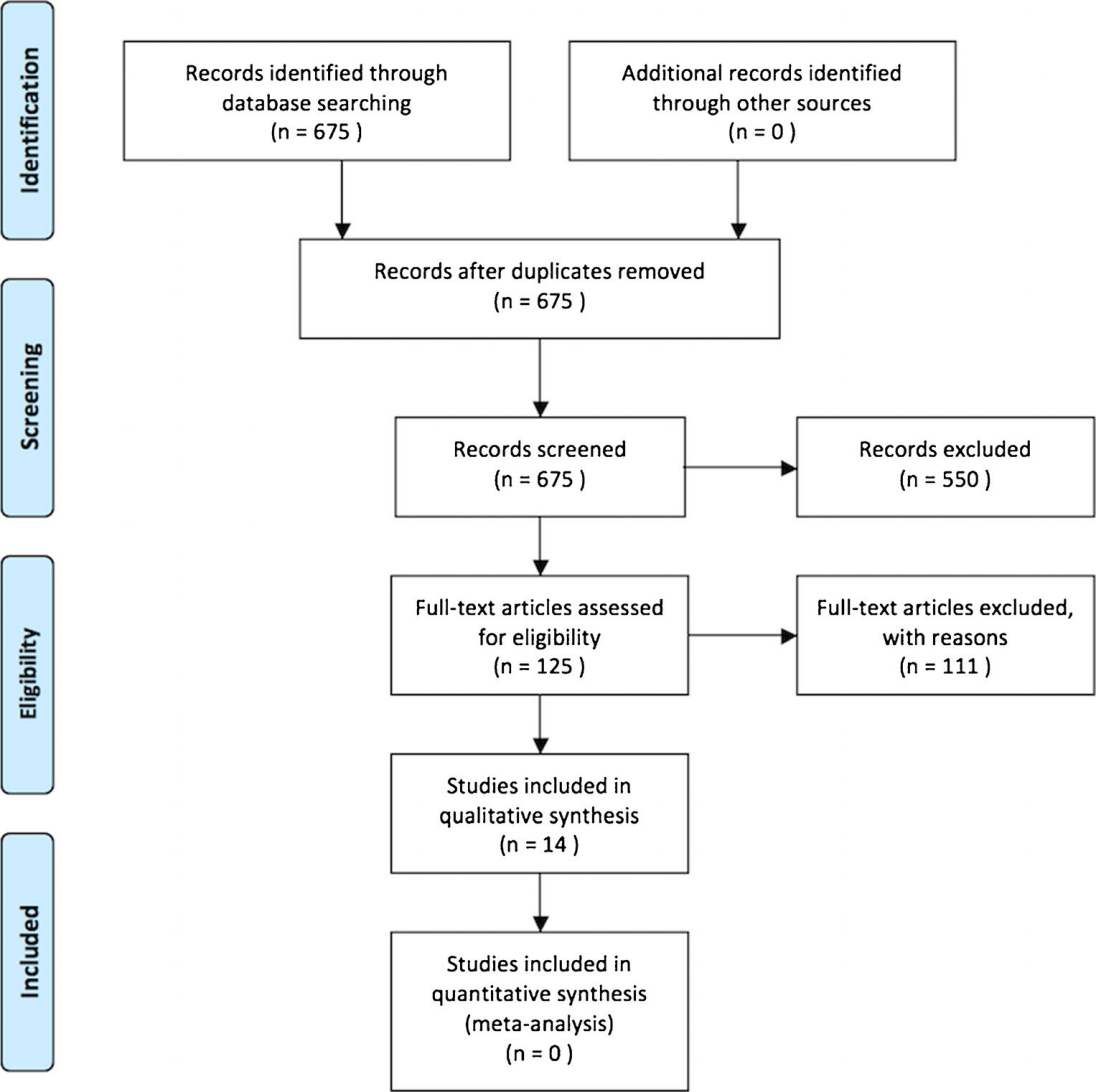
PRISMA flow chart for the systematic review

The PUBMED search was performed 21 April 2015 and yielded 675 potential articles. Limiting to human studies excluded 96, limiting to English language excluded a further 189 and excluding case reports excluded a further 265, leaving 125 articles. Application of the exclusion criteria to the electronic abstract excluded 111 of these, leaving 14 articles included in the systematic review. The PRISMA flow chart is shown in Fig. 1.

The exclusion log recorded that of the 111 excluded articles, 65 were primarily because they did not describe MP, 17 were narrative reviews, two were letters, nine described no cross-sectional imaging, five were ‘small’ by our definition, one recruited malignancy exclusively and 12 were published prior to 1973. We found no systematic reviews.

### Study characteristics and design

Characteristics for included studies are described in Table 1. Overall, included studies contributed 1,226 patients with MP, with an individual range between 17 and 359. Median patient age was 62 years, range 11–98. Of studies stating gender, 603 (70 %) patients were male and 264 (30 %) were female. Females predominated in a single study [17]. Only three (21 %) studies accrued patients prospectively [1, 17, 20], whereas ten were retrospective (71 %). One combined retrospective and prospective accrual [15]. None were genuinely multicentre.

**Table 1:** Characteristics of included studies and patients

Individual study	Patient accrual	Multi- or single-centre	No. of patients	Male/female	Mean age (range)
Akram [15]	Mixed	Single	92	64/28	64.5 (55–72)
Badet [16]	Retrospective	Single	158	121/37	63 (27–98)
Canygit [2]	Retrospective	Single	51	35/16	56.2 (33–78)
Corwin [6]	Retrospective	Single	37	22/15	62.6 (25–90)
Coulier [1]	Prospective	Single	48	29/19	not stated
Daskalogiannaki [17]	Prospective	Single	49	17/32	62 (27–84)
Gogebakan [12]	Retrospective	Single	77	59/18	65.5 (SD 11.9)
Nakatani [18]	Retrospective	Single	71	47/24	65 (39-88)
Roson [19]	Retrospective	Single	26	18/8	69 (35–85)
Sabate [8]	Retrospective	Single	17	14/3	52 (11–84)
Seo [20]	Prospective	Single	29	19/10	57 (18–85)
Smith [10]	Retrospective	Single group	359	not stated	66.9 (19–97)
van Putte-Katier [3]	Retrospective	Single	94	66/28	66.6 (SD 11.2)
Wilkes [11]	Retrospective	Single group	118	92/26	61 (20–88)

*SD* standard deviation

Twelve (86 %) studies focused on CT features of MP. One described positron emission tomography (PET)-CT [18] while another described ultrasound [19]. Diagnosis of MP was established histologically in all patients in two studies [8, 15]. Histology was used in an unspecified proportion of patients in four other studies [10, 17, 18, 20], but most relied on CT/imaging features themselves to establish the diagnosis. In studies describing a follow-up period, this ranged widely, varying from less than 1 month [20] to 8 years [19] for individual patients.

Seven studies stated the total population of cases from which cases of MP were drawn, allowing calculation of prevalence of abnormality. Two [11, 12] of these used a retrospective keyword search to identify patients whereas five [1–3, 17, 18] interrogated a consecutive patient series. Overall, prevalence of MP for studies using a keyword search was 0.2 % (195 of 87,017 patients), compared to 1.7 % (313 of 18,389) for studies using consecutive series.

We concluded that spectrum bias influencing patient accrual was potentially present in at least nine (64 %) of the 14 studies. This was mostly because accrual was retrospective and relied upon identifying patients via a keyword search of the radiology information system. This approach will miss patients if the precise term does not appear in the report or those in whom the condition is not reported [6, 8, 10–12, 16, 19]. One study only recruited patients with a histological diagnosis, thus biasing accrual towards severe cases [15], whereas another recruited only patients having PET-CT, inducing bias towards patients with malignancy [18]. One study used a case-control design, matching MP patients with two age- and gender-matched controls [12]. Five studies reduced selection bias by reporting consecutive case series. Two [2, 3] of these were retrospective and three [1, 17, 20] were prospective. Unfortunately, the prospective studies lacked details of subsequent malignancy[20], were hampered by incomplete follow-up [17] or were restricted to patients having subsequent CT [1]. Overall, we concluded there were just four studies whose design allowed the reader to determine the proportion of an unselected consecutive group of patients with MP who developed malignancy subsequently. However, three of these were retrospective and potentially subject to accrual bias from keyword searching [6, 10, 19]. This left just one study in which consecutive unselected patients were analysed, but this was retrospective, single centre and had excluded any patients with a nodal diameter in excess of 1 cm, a procedure likely to exclude some patients with idiopathic MP [3].

### Associated symptoms and malignancy

Only eight (57 %) studies described the proportion of symptomatic patients: 710 (79 %) of 894 patients were symptomatic, with individual proportions ranging from 23 % [19] to 100 % [8, 10, 17, 21]. ‘Abdominal pain’ was the most frequent description reported, occurring in a median of 51 % of patients in whom symptoms were reported (range 6–83 %). Other symptoms were reported too variably to summate but included abdominal distension and change in bowel habit.

Nine studies described surgical history but five of these either did not detail the specific procedure or did not quantify them. Of those detailing surgical history, 270 (43 %) of 634 patients had prior surgery; the individual proportion ranged from 31 % [19] to 57 % [17]. The most common procedures were cholecystectomy or appendectomy.

Thirteen studies described the proportion of patients with known malignancy at the time of MP diagnosis. Of 1,209 patients overall, 458 (38 %) had known malignancy. This proportion ranged widely for individual studies; from 8 % [6] to 89 % [18], precluding meta-analysis. Of these 458, 115 (25 %) were lymphomas and 343 (75 %) were other malignancies. Again, the proportion of patients with lymphoma varied widely for individual studies, from 3 % [15] to 34 % [18]. In eight studies it was possible to calculate the proportion of patients who developed a new malignancy subsequent to diagnosis of MP: This occurred in 60 (6.4 %) of a total 933 patients. Of these 60 malignancies, 20 (33 %) were lymphomas and 40 (67 %) were other malignancies. The proportion of patients developing a new malignancy varied from 0 % [17, 19] to 11 % [11] for individual studies.

### Imaging features of mesenteric panniculitis

We investigated eight CT features of MP but we were unable to extract all of these from any individual article, indicating that description of CT features was generally poor. Of the nine studies that described whether patients had increased mesenteric density on CT scanning, 556 (90 %) of 617 patients displayed this feature. Indeed, eight studies stipulated this feature as an inclusion criterion [1–3, 6, 8, 16–18]. A discrete ‘mass’ was described in 473 (93 %) of the 509 patients in whom this was evaluated. Again, several studies stipulated this feature as an inclusion criterion [1–3, 8, 16, 17]. Only four (27 %) studies documented the dimension of any mass, reporting means of 40 mm (range 20–74) [20], 50 mm (27–108 mm) [2], 95 mm (range 71–152 mm) [17] and 95 mm (standard deviation 19 mm) [3], respectively. Six studies [1–3, 8, 16, 17] stated whether a hyperattenuating rim appeared to encapsulate the region of mesenteric panniculitis, this feature being present in 239 (57 %) of the 417 patients evaluated. The individual proportion ranged between 35 % [8] and 59 % [16, 17].

Given that lymph nodes are a prominent feature of mesenteric panniculitis [13], the description of nodal imaging characteristics was surprisingly sparse overall. Some studies described ‘bulky lymphadenopathy’ but did not define the nodal diameter or other characteristics necessary to satisfy this definition [20]. Others defined lymphadenopathy as a diameter greater than 1 cm and noted the proportion of patients exceeding this threshold, but gave no other details; for example the mean diameter and range of nodal size for these patients or for patients overall [8]. Conversely, one study excluded patients with nodes measuring 1 cm or greater, believing this to exclude the diagnosis of mesenteric panniculitis [3]. The largest nodal diameter could be determined in only four (27 %) studies, being 13 mm [2], 19 mm [1], 35 mm [6] and 41 mm [12], respectively. Ultimately, only two studies described mean nodal diameter and the range across all patients, this being 6.4 mm (range 3.1–13 mm) [2] and 8.7 mm (range 3–35 mm) [6], respectively. Seven studies [1–3, 8, 16, 17, 20] stated whether lymph nodes were surrounded by a hypoattenuating ‘halo’ within the region of mesenteric panniculitis, a feature present in 284 (64 %) of the 446 patients in whom this was assessed. The individual proportion ranged widely between 7 % [20] and 94 % [3].

## Discussion

Mesenteric panniculitis presents a clinical problem when discovered incidentally in patients not known to have an underlying cause, in which case it can be termed ‘idiopathic’. Because an association with subsequent malignancy has been described, patients are frequently referred for follow-up imaging on multiple occasions over a prolonged period. The precise extent to which this happens is unknown currently, but anecdotal evidence suggests it is substantial, not least because features of MP often persist. We were prompted to perform our systematic review because this set of circumstances had arisen on multiple occasions during our hospital multidisciplinary meetings. In particular, we were interested in assessing primary research evidence linking apparently idiopathic MP with subsequent malignancy.

We found that 38 % of reported patients had known underlying malignancy at the time MP was diagnosed. In such cases MP assumes a minor role, since attention will focus on known malignancy. Studies that include patients with known malignancy will confound any attempt to determine the clinical course of patients with incidental MP. The true incidence of MP is difficult to determine since this requires assessment of a consecutive, unselected group of patients by radiologists aware of and able to diagnose the condition. Most studies applied a search term to a radiology information system retrospectively, potentially missing patients whose report did not contain the term. One study [3] used a retrospective cohort design to interrogate consecutive scans, which eliminates inclusion bias, but then excluded patients with lymphadenopathy, which will likely eliminate some patients with idiopathic MP. A minority did report consecutive, prospective cases but were often personal series hampered by small size and single-centre accrual. We found the prevalence of MP was just 0.2 % for studies using a retrospective keyword search versus 1.7 % for studies interpreting a consecutive series. These data suggest that keyword searches miss most patents with MP.

Severe manifestations of MP do appear to exist, but the overall proportion of patients with proven histology was very small, suggesting the large majority do not come to surgery for MP alone. Stipulating histological proof of MP would create substantial spectrum bias towards severe disease, unrepresentative of most patients with MP. While, strictly speaking, the true prevalence of idiopathic MP can only be ascertained by examining asymptomatic patients as well, the clinical problem turns on imaged patients who then present a clinical dilemma. Accordingly, the majority of studies used CT scanning as the reference standard for diagnosis. Although this suggests incorporation bias, we do not believe this is a major issue because the clinical issue revolves around these very same patients. CT scans on which MP is diagnosed are usually performed to investigate abdominopelvic symptoms, so we would expect symptom prevalence to appear high: Overall, 79 % had symptoms. However, it is unclear what proportion of idiopathic MP is symptomatic. For example, many patients had underlying malignancy and/or symptoms that may be unrelated to MP. Our anecdotal experience in patients without malignancy suggests that symptoms precipitating the initial scan often subside but features of MP persist across subsequent imaging. Unfortunately, individual studies reported insufficient detail for us to resolve these issues. Ultimately, we concluded that the incidence of idiopathic MP is unknown with precision currently.

Our primary research question asked what proportion of patients with idiopathic MP then develop subsequent malignancy? However, many studies did not report a follow-up period, or this was relatively short. Unfortunately, we concluded that just four studies allowed the reader to determine the proportion of an unselected consecutive group of MP patients who developed malignancy subsequently. Ultimately, because three of these were potentially subject to accrual bias and the fourth had excluded patients with lymphadenopathy in excess of 1 cm, we concluded that no study in the existing literature could answer our research question using sound methodology. Our intention to perform meta-analysis was frustrated. Furthermore, we found no prior systematic reviews. Ultimately, we do not know whether patients with apparently idiopathic MP are at greater risk of subsequent malignancy than the age- and sex-matched general population, a conclusion also reached by a recent commentary [22].

A research study to answer this question should recruit consecutive, unselected patients prospectively, to reduce inclusion bias. Patients should be representative of those undergoing abdominopelvic CT for all indications since CT is the modality usually precipitating the problem. Observers should be radiologists familiar with and able to diagnose MP, and precise CT definitions for the diagnosis should be stipulated (which was largely lacking from the papers we identified). Patients would need to be characterised in detail, to identify and exclude those with existing or undiagnosed malignancy at the time of imaging, and to clarify symptoms and their natural history. Follow-up should be sufficient to establish clinical outcome with precision, and should extend several years to determine any link with subsequent malignancy, likely via cancer registries so as to maximize data capture and to allow comparison with age- and sex-matched controls. A multicentre setting would be necessary both to achieve sufficient power and to enhance generalisability of results. Imaging findings should be correlated with clinical outcome. For example, we attempted to extract eight individual CT features of MP but could not do this completely for any individual article. Indeed, several studies failed to report the imaging features of MP or characterise lymphadenopathy. Description of the CT features of MP is therefore generally poor, which hinders any critical discussion of the precise imaging features necessary for diagnosis.

In summary, we performed a systematic review to determine any association between imaging findings of idiopathic MP and development of subsequent malignancy. Overall, studies were heterogeneous and used varying methodologies to identify patients, resulting in biased accrual. Frequently, patients and their clinical follow-up were characterised insufficiently to answer our research question. We could not perform meta-analysis. Any association between imaging features of idiopathic MP and development of subsequent malignancy remains unknown presently. The available literature currently lacks studies that are methodologically sound enough to answer this important question with precision.
